# Predictions of the Biological Effects of the Main Components of Tarragon Essential Oil

**DOI:** 10.3390/ijms26051860

**Published:** 2025-02-21

**Authors:** Andrijana Pujicic, Iuliana Popescu, Daniela Dascalu, David Emanuel Petreuș, Adriana Isvoran

**Affiliations:** 1 Department of Biology, West University of Timisoara, 16 Pestalozzi, 300115 Timișoara, Romania; andrijana.pujicic@e-uvt.ro; 2 Faculty of Agriculture, University of Life Sciences “King Mihai I” from Timisoara, 119 Calea Aradului, 300645 Timișoara, Romania; iuliana_popescu@usvt.ro; 3 Department of Chemistry, West University of Timisoara, 16 Pestalozzi, 300115 Timișoara, Romaniadavid.petreus03@e-uvt.ro (D.E.P.)

**Keywords:** ADMET profile, antibacterial activity, cytotoxicity, regulator of G-protein signaling 17

## Abstract

*Artemisia dracunculus*/(tarragon) is a perennial plant used in traditional medicine and the food industry. The plant is known to have beneficial effects on health, such as antibacterial, antifungal, antiseptic, carminative, anti-inflammatory, antipyretic, anthelmintic, etc. In this study, the compounds present in the highest concentrations in the essential oils obtained by different extraction methods from tarragon found on the Romanian market were identified by gas chromatography–mass spectrometry. The biological activity of these compounds was predicted using the computational tools ADMETlab3.0, admetSAR3.0, CLC-Pred2.0, and AntiBac-Pred. Also, the main molecular target of these compounds was predicted and the interactions with this protein were evaluated using molecular docking. The compounds identified in high concentrations in the obtained essential oils are estragole, cis-β-ocimene, trans-β-ocimene, limonene, eugenol methyl ether, eugenol acetate, eugenol, caryophyllene oxide, and α-pinene. The absorption, distribution, metabolism, excretion, and toxicity profiles of these compounds show that they are generally safe, but some of them can cause skin sensitization and respiratory toxicity and are potential inhibitors of the organic anion transporters OATP1 and OATP2. Several of these compounds exert antibacterial activity against some species of *Staphylococcus*, *Streptococcus*, and *Prevotella*. All compounds reveal potential cytotoxicity for several types of cancer cells. These findings may guide further experimental studies to identify medical and pharmacological applications of tarragon extracts or specific compounds that can be isolated from these extracts.

## 1. Introduction

The herbal extracts market has grown significantly in recent years, driven by the growing consumer demand for herbal products, due to the growing awareness of the therapeutic properties of these extracts. The global herbal extracts market was valued at USD 40.1 billion in 2020 and is expected to grow to USD 78.1 billion by 2032 [1]. In Europe, the herbal extracts market was valued at approximately USD 3.91 billion in 2024 and is expected to grow to USD 6.11 billion by 2029 [2]. Likewise, it has been observed that 80% of the world’s population chooses traditional medicine instead of conventional medicine for health care [3], because, due to their natural origin, herbal products are considered safe. Furthermore, specific studies in the literature report that the overconsumption of chemical preservatives from food products may lead to health issues and there is a need to search for novel antibacterial and antifungal agents, such as those originating from natural sources [4]. However, there is a lack of studies on the efficacy and toxicity of plant extracts, as they may contain biologically active ingredients producing adverse effects that should be considered and evaluated [5,6,7]. It underlines the necessity of implementing studies for assessing both beneficial and adverse effects of the compounds found in natural extracts.

Tarragon (*Artemisia dracunculus*) is a perennial herb in the Asteraceae family. Its aerial part and/or leaves are used for culinary purposes, especially in Europe, by enhancing the flavor of many dishes or as food preservatives [8,9,10]. There also are traditional medicinal benefits associated with its use, mainly known in Asian countries. Tarragon, and especially tarragon essential oil, are known to have anticoagulant, anti-epileptic, anti-inflammatory, anti-hyperglycemic, antimicrobial, antifungal, antioxidant, and antispasmodic properties; it is used as an appetite stimulant, digestive aid, sleep aid, pain relief, and for the treatment of cardiovascular disease and thrombosis [8,10,11,12,13,14,15]. Consequently, the tarragon market size is predicted to increase by USD 127.4 million by 2028 [16].

Fewer side effects of tarragon essential oil are known. Animal studies have shown mutagenic and carcinogenic properties of tarragon essential oil, which may be due to the content of estragole or other mutagenic compounds [17,18]. Tarragon compounds such as coumarins may enhance the blood-thinning effect and may interact with anticoagulant drugs and increase the risk of bleeding [19]. Plants of the Asteraceae family are known to produce allergic reactions [20]; so, tarragon may also be responsible for such reactions.

The beneficial and the toxicological effects of tarragon are due to its chemical composition. The chemical compounds identified in tarragon essential oils were not always the same and the differences might be related to geographical situation, ground conditions, harvest time, the age of the plant, genetic factors, or extraction methods [12,15]. The main tarragon compounds that have been identified in numerous studies are α-ocimene, β-ocimene, α-pinene, elemicin, estragole (methyl chavicol), eugenol, geranial, limonene, linalool, terpinen-4-ol, and sabinene [12,15,21]. Small doses of these compounds resulting from the culinary use of tarragon are generally considered safe, but the long-term or high-dose use of tarragon or its essential oil may have toxic effects, as there are studies revealing the toxicity of some of the identified compounds. β-ocimene may produce skin and eye irritation [22] and was predicted to be able to produce respiratory toxicity, hepatotoxicity, mutagenicity, and carcinogenicity [23]. The European Food Safety Authority (EFSA) classified methyl eugenol and estragole as genotoxic and carcinogenic compounds in rodents [24]. Geranial was predicted to produce respiratory toxicity and to have carcinogenic potential [23]. Several of these compounds are considered as being able to penetrate the blood–brain barrier and to have effects on the central nervous system or to interact with cytochromes that are involved in metabolism of drugs or endobiotics [23].

Even though tarragon is considered a medicinal plant with various bioactive compounds and has been studied for its anticancer, antimicrobial, and anti-inflammatory potential, available data on the absorption, distribution, metabolism, excretion, and toxicity (ADMET) profiles of compounds found in tarragon essential oil are not widely reported. The aim of this study is to evaluate the ADMET profiles of bioactive compounds identified in higher concentrations in essential oils of tarragon obtained by different extraction methods from tarragon found on the Romanian market by using a computational approach. These predictions help researchers understand how these compounds might be absorbed and distributed, how they might be metabolized in the body, and whether they have the potential to cause toxicity.

## 2. Results

### 2.1. Identification of Volatile Compounds in Tarragon Extracts

Compounds identified in the obtained tarragon essential oils by using the gas chromatography–mass spectrometry method are revealed in Table 1. Corresponding chromatograms are given in Supplementary Figures S1–S4.

**Figure 1:**
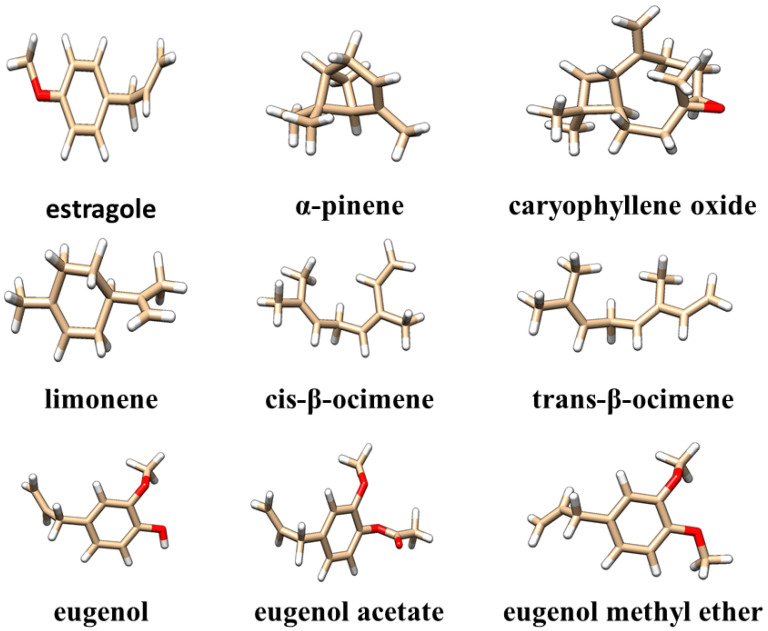
Three-dimensional structures of the main compounds identified in the obtained tarragon essential oils. Compounds are visualized as colored sticks: carbon—brown, oxygen—red, and hydrogen—white.

The data presented in Table 1 show that oxygenated monoterpenes are the predominant class of chemicals in tarragon essential oils. Of these, estragole (Figure 1) is the compound found in the highest concentration, regardless of the method used to obtain the tarragon essential oil. Other compounds found in concentrations greater than 1% in at least one of the obtained tarragon essential oils are as follows: cis-β-ocimene, trans-β-ocimene, caryophyllene oxide, limonene, eugenol, eugenol acetate, methyl eugenol ether, and α-pinene (Figure 1). The structural files of these compounds were extracted from the PubChem database [26] and visualized using Chimera1.16 software [27].

The identified compounds in the essential oils are dependent on the extraction method, as each method selectively targets different bioactive compounds based on their chemical properties. For similar compounds that are identified in essential oils from extracts obtained by distinct methods, their concentrations are distinct. Furthermore, in the case of essential oils from the extracts obtained by hydrodistillation, there are distinct concentrations for the compounds identified in the essential oils from the extracts obtained from dried and fresh tarragon, respectively. It is not an unexpected result, as fresh tarragon has a higher water content, which can influence the volatility and extraction efficiency of the essential oils; the high water content competes with the essential oils during hydrodistillation.

### 2.2. ADMET Profiles of the Main Compounds of Tarragon

The physicochemical properties and the ADME profiles of the compounds found in higher concentrations in tarragon essential oils and obtained using admetSAR3.0 [28] and ADMETLab3.0 [29] are presented in Supplementary Materials, Tables S1–S6. The physicochemical properties of these compounds reveal that all compounds have low molecular weights, a small number of hydrogen bond donors and acceptors, a reduced number of rotatable bonds, and a hydrophobic character (Supplementary Materials, Table S1). All compounds meet the Lipinski rule and are considered to have good oral bioavailability, but α-pinene, cis-β-ocimene, trans-β-ocimene, and limonene do not satisfy the Pfizer and GSK rules, and caryophyllene oxide does not satisfy the Pfizer rule (Supplementary Materials, Table S2). This indicates potential safety concerns. It underlines the necessity to obtain safety information regarding the main compounds found in tarragon essential oil.

The results obtained using admetSAR3.0 and ADMETLab3.0 tools regarding the absorption and distribution profiles of the compounds found in tarragon essential oils (Supplementary Materials, Tables S3 and S4) reveal that all the compounds have a good human intestinal absorption. In the case of distribution profiles, both computational tools predict that compounds identified in tarragon essential oils are able to inhibit the organic anion transporter peptides OATP1 and OATP2, and that these compounds reversibly bind to plasma proteins. However, the other predictions are quite distinct. The admetSAR3.0 predicts that all the investigated compounds are able to cross the blood–brain barrier (BBB), but predictions obtained using ADMETLab3.0 indicate that limonene has a good ability to cross the barrier, and eugenol acetate and cariophyllene oxide exhibit low probabilities of crossing the blood–brain barrier. Similarly, admetSAR3.0 predicts that investigated compounds are not inhibitors of P-glycoprotein (P-gp), whereas ADMETLab3.0 indicates that, except for limonene and eugenol, the other compounds reveal at least low probabilities of inhibiting P-gp. These inconsistences are discussed further (see Section 3).

According to the predictions obtained with admetSAR3.0, none of the compounds are considered as inhibitors of the breast cancer resistance protein (BCRP), bile salt export pump (BSEP), and multidrug and toxin extrusion protein 1 (MATE1). On the other hand, the outcomes of ADMETLab3.0 indicate that, except for α-pinene, limonene, and caryophyllene oxide, the other compounds reveal at least small probabilities to be inhibitors of BCRP, with eugenol and eugenol methyl ether having the highest probabilities to inhibit this protein. This prediction is in good correlation with the ability of these compounds to produce cytotoxicity against breast cancer cells (see further). α-pinene, cis-β-ocimene, trans-β-ocimene, limonene, and caryophyllene oxide are the compounds with reasonable probabilities to inhibit multidrug resistance protein 1 (MRP1) and, except for cis-β-ocimene, all the other compounds may inhibit BSEP.

Predictions of the metabolism of compounds identified in tarragon essential oil were obtained using the two computational tools, and the results are also quite distinct (Supplementary Materials, Tables S5 and S6). Conforming to results obtained using admetSAR3.0, caryophyllene oxide presents a high probability to be an inhibitor of CYP2B6, and estragole, α-pinene, cis-β-ocimene, trans-β-ocimene, and limonene also have reasonable probabilities to be inhibitors of this cytochrome. Furthermore, CYP1A2 may be inhibited by estragole, eugenol, eugenol acetate, and eugenol methyl ether. Conforming to results obtained using ADMETLab3.0, CYP1A2 may be inhibited with high probability by eugenol and eugenol acetate, and with low probability by limonene and eugenol methyl ether. Eugenol, eugenol acetate, and eugenol methyl ether are also considered as inhibitors of CYP3A4, CYP2B6, and CYP2C9, and estragole is considered as an inhibitor of CYP3A4. All compounds may be inhibitors of CYP2C19.

The predictions made by both computational tools concerning the plasmatic clearance indicate that all the compounds are being eliminated in a moderate rate from the bloodstream.

The toxicity of the identified compounds against the human organism is presented in Table 2 and Table 3. Table 2 reveals the information obtained using the admetSAR3.0 computational tool and Table 3 reveals the result of the ADMETLab3.0 tool.

The results obtained using both the admetSAR3.0 and ADMETLab3.0 tools show that none of the investigated compounds are considered to produce neurotoxicity, hematotoxicity, and reproductive toxicity, and there are usually low probabilities that these compounds produce cardiotoxicity and mutagenicity. Also, the results obtained using both tools show that all compounds can induce skin sensitization (high probabilities) and nephrotoxicity (usually low probabilities).

The results obtained using ADMETLab3.0 illustrate that these compounds do not cause hepatotoxicity but can produce respiratory toxicity and, with the exception of eugenol acetate and trans-β-ocimene, there are low probabilities that these compounds produce carcinogenicity.

### 2.3. Antibacterial Activity of the Main Compounds of Tarragon Essential Oil

Traditional medicine considers tarragon to have antimicrobial properties. In the present study, the antibacterial properties of the main compounds identified in tarragon essential oils were predicted using AntiBac-Pred [30], and the results are presented in Table 4. Compounds that are not present in Table 4 were predicted to have antibacterial properties with less than 0.5 confidence.

The data presented in Table 4 show that some of the compounds identified in tarragon essential oils may be effective against several types of microorganisms, including *Staphylococcus*, *Streptococcus*, and *Prevotella* species.

### 2.4. Cytotoxicity of the Main Compounds of Tarragon Essential Oils

Although tarragon’s use in treating cancer is well-embedded in traditional medicine, modern scientific research on the anticancer properties of tarragon essential oil is limited. It has been proved that terpenoids, the class of compounds that were mainly identified in the tarragon essential oils used in the present study, possess anticancer potential [31,32] through mechanisms such as inducing apoptosis, inhibiting tumor cell migration, and modulating pathways involved in cell proliferation due to their ability to modulate enzymes involved in cancer development [33]. There also is currently a great interest in identifying natural bioactive compounds possessing anticancer properties. Consequently, the cytotoxicity of compounds found in tarragon essential oils was analyzed in this study. The results of the CLC-Pred2.0 computational tool [34] are disclosed in Table 5. Only predictions with a probability greater than 0.5 that the compounds are active against cells were considered, except in the case of caryophyllene oxide, where the predicted molecular target with the highest probability, 0.276, was the regulator of G-protein signaling 17.

The molecular mechanisms by which tarragon essential oils exert cytotoxic effects on cancer cell lines are not known. Consequently, in the present study, the molecular targets of the compounds identified in tarragon essential oils together with the type of effects they may have on that target were predicted and are also revealed in Table 5.

The data presented in Table 5 show that compounds found in higher amounts in the obtained tarragon essential oils are thought to produce cytotoxicity against numerous types of cancer cells. Furthermore, predictions show that all compounds produce toxicity on cisplatin-resistant ovarian carcinoma cells. Among the compounds identified, caryophyllene oxide appears to be active against most types of cancer cells. These predictions also reveal that all the compounds that were identified in tarragon essential oils in the present study are able to inhibit the regulator of G-protein signaling 17 (RGS17).

### 2.5. Molecular Docking Study Concerning the Interactions of the Compounds Found in Tarragon Essential Oils with the Regulator of G-Protein Signaling 17

**Figure 2:**
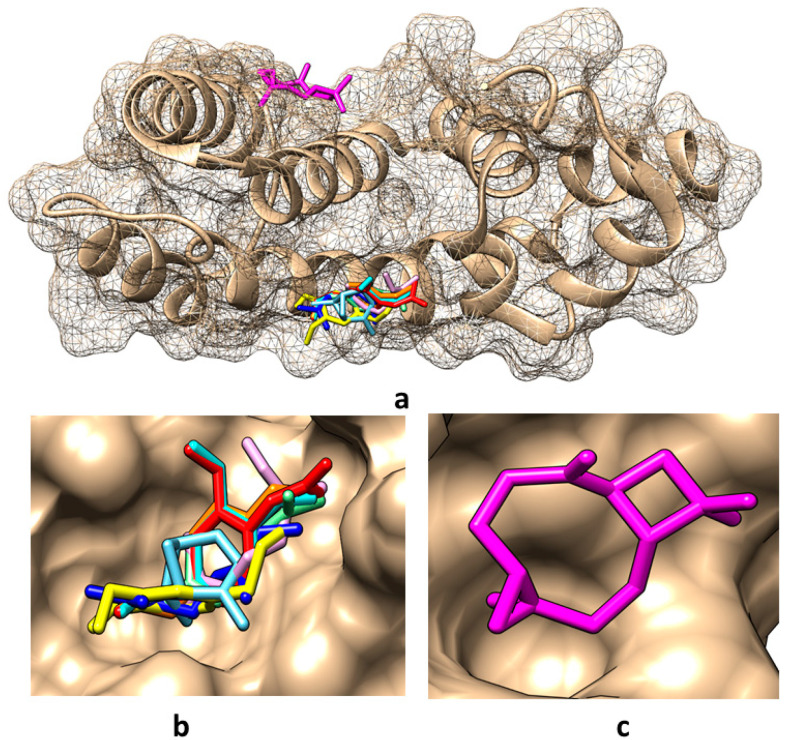
Best binding modes for the main compounds identified in obtained tarragon essential oils to RGS17 protein when using the structural file 1ZV4: (**a**) overall view; (**b**) detailed view for binding of α-pinene, cis-β-ocimene, trans-β-ocimene, limonene, estragole, eugenol, eugenol acetate, and eugenol methyl ether; (**c**) detailed view for binding of caryophyllene oxide. Color representation: α-pinene—light blue, caryophyllene oxide—magenta, cis-β-ocimene—light purple, trans-β-ocimene—dark blue, limonene—light green, estragole—orange, eugenol—yellow, eugenol acetate—red, and eugenol methyl ether—cyan.

**Figure 3:**
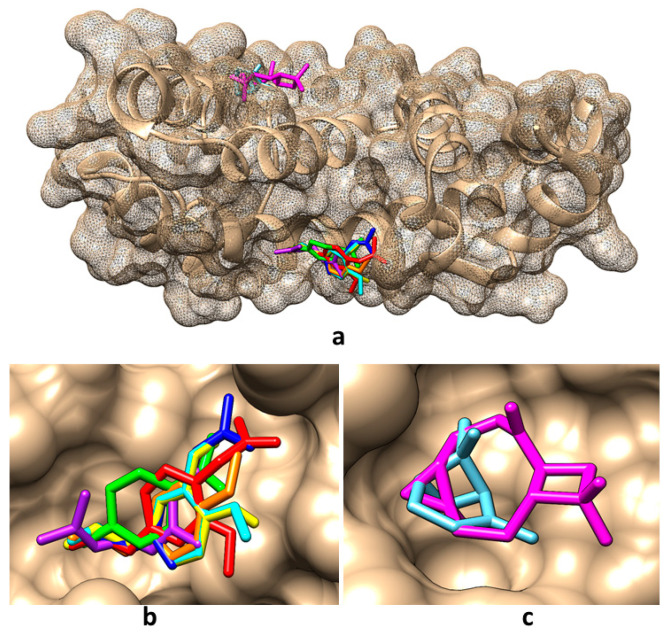
Best binding modes for the main compounds identified in obtained tarragon essential oils to RGS17 protein when using the structural file 6AM3: (**a**) overall view; (**b**) detailed view for binding of cis-β-ocimene, trans-β-ocimene, limonene, estragole, eugenol, eugenol acetate, and eugenol methyl ether; (**c**) detailed view for binding of α-pinene and caryophyllene oxide. Color representation: α-pinene—light blue, caryophyllene oxide—magenta, cis-β-ocimene—light purple, trans-β-ocimene—dark blue, limonene—light green, estragole—orange, eugenol—yellow, eugenol acetate—red, and eugenol methyl ether—cyan.

Taking into account the predictions obtained in the present study regarding the molecular targets of the compounds found in the obtained tarragon essential oils, the molecular docking method has been considered to analyze the potential interactions between these compounds and RGS17. The Protein Data Bank (PDB) [35] contains two crystallographic structures of RGS17: (i) the structural file 1ZV4 of the native protein [36] and (ii) the structural file 6AM3 of the RGS17 co-crystallized with Ca^2+^ that is bound to conserved positions on the predicted Gα-binding surface of the protein [37]. Both structures have been considered for implementing molecular docking; the interacting energies and the amino acids involved in the interactions are revealed in Table 6. Pictures illustrating the best binding modes for the compounds found in tarragon essential oils are revealed in Figure 2 and Figure 3.

Data presented in Table 6 indicate that all the investigated compounds are moderately favorably interacting with RGS17 by hydrophobic interactions and there are only small differences in the interacting energies obtained in the absence and presence of calcium ions. This means that the compounds found in tarragon essential oils may act as moderate inhibitors of RGS17, potentially having clinical utility as chemotherapeutics in the treatment of several types of cancers.

Figure 2 and Table 6 show that in the absence of calcium ions, except for caryophyllene oxide which binds in the region of amino acids LEU115, ALA116, ASP119, MET135, ILE136, and TYR140, all other compounds bind in the region of amino acids TRP114, GLN175, LEU176, TYR179 of RGS17. When calcium ions are present, α-pinene and caryophyllene oxide bind in the region of amino acids ALA116, ASP119, LYS132, MET135, and ALA136, and all other compounds bind in the region of amino acids TRP114, GLN175, and LEUR176 (Figure 3). In addition, among the investigated compounds, α-pinene and caryophyllene oxide reveal the lowest affinity for the RGS17 and eugenol, and eugenol acetate and limonene reveal the highest affinities for RGS17, being potentially better inhibitors of this protein.

Further experimental studies are needed to confirm the predicted anticancer properties, but these results reveal that tarragon essential oil may be used as an anticancer agent for the treatment of several types of cancer as an adjunct remedy for patients treated with chemotherapy, as it may allow for lower doses of chemotherapy, resulting in a boosted effectiveness with decreased toxicity.

## 3. Discussion

### 3.1. Comparison Between the Computational Tools Used to Obtain the ADMET Profiles of Compounds Identified in Tarragon Essential Oils

In this study, we considered two computational tools for predicting ADMET profiles of compounds identified in tarragon essential oils, ADMETLab version 3.0 and admetSAR version 3.0. These tools were considered because they integrate vast datasets containing information from multiple types of chemicals, allowing for accurate predictions of various ADMET properties with correlation coefficients usually exceeding 0.80 [28,29]. Last but not least, their friendly interface and ease of use, in addition to their continuous updates, make them reliable tools for ADMET profiling. ADMETLab and admetSAR tools were widely used in predicting the ADMET profiles of various types of chemicals, particularly in the fields of drug discovery [38] but also for safety assessments of various types of chemicals [23,39,40,41,42,43,44]. The differences between the two tools are highlighted in the following.

The admetSAR tool is more centered on providing a broad database of known compounds and their associated ADMET data and allows users to perform predictions that are usually based on the quantitative structure–activity relationship (QSAR) and statistical models. This computational tool has been widely used in research (admetSAR1.0 version was cited 2088 times and admetSAR2.0 was cited 1130 times) and has a good reputation for its ability to predict ADMET profiles but may perform less well for compounds that are not well represented in the training datasets [28]. With the exception of eugenol, whose properties fully correspond to the applicability domain (the chemical space in which predictive models are reliable) in the admetSAR3.0 tool, the other compounds are only 50% similar to the compounds in the training datasets (Supplementary Materials, Table S2). This indicates that predictions should be treated with caution, with further experimental validation being necessary.

ADMETLab3.0 uses sophisticated machine learning algorithms and advanced statistical techniques to make predictions. Regarding the present study, all the investigated compounds usually fall within the Upper Limit Domain, the model’s predictions being considered reliable because the compound’s characteristics fall within the range of compounds the model was trained on. Consequently, the results obtained using ADMETLab3.0 may usually provide higher accuracy in the case of these compounds, both due to the fact that the investigated compounds fall within the upper limit range and due to the use of newer technologies in predictions, such as deep learning, reflecting the diversity of its models. In addition, ADMETLab3.0 is able to distinguish between cis-trans isomers due to its ability to analyze and learn from chemical and topological descriptors specific to each isomer [29]. Another study on the ADMET profiles of the stereoisomers of difenoconazole (a fungicide used in agriculture) also revealed that the models integrated in the ADMETLab tool were more suitable for isomer screening compared to the models incorporated in the admetSAR tool [39].

However, the reliability of predictions obtained using the admetSAR and ADMETLab tools depends on the quantity and quality of data used to train the models and the diversity of chemicals included in the training datasets, which can introduce biases that affect the predictions. Biases in datasets can result from poor data quality (data that were obtained in poor experimental designs or measurement errors), from limited biological data, or from the fact that models obtained in laboratory animals do not generalize well to humans. Another bias may come from the fact that the dataset predominantly includes compounds with drug-like properties, and other classes of chemicals, such as natural products, may not be accurately predicted. Consequently, even if the reliability of predictions is generally high, it is important to remain cautious when applying these predictions to natural compounds. These potential biases when using these tools for chemical screening should be taken into account and the validation of predictions with experimental data is necessary to ensure the safety and efficacy of natural compounds.

Computational models, particularly those used in drug discovery and ADMET predictions, have made significant advances, but they still face challenges, especially when handling stereochemistry and complex metabolic pathways. Many computational models rely on simplified representations of molecules (usually 2D molecular graphs) that do not completely capture the 3D spatial orientation of stereoisomers, and this can lead to incorrect predictions about how a molecule will interact with biological targets [45]. The available data on chiral centers may be insufficient for robust training of models and the stereoisomeric effects may not be fully considered in predictions. Furthermore, metabolic pathways can be highly complex, involving numerous enzymes with a high flexibility and broad specificity, and cellular interactions and computational models may face difficulties in accurately modeling these processes. Even with limitations in handling stereoisomers and complex metabolic pathways, computational methods are continually being improved to overcome these difficulties. These methods are crucial tools in many areas of pharmaceutical research by complementing experimental approaches, allowing researchers to make informed decisions, saving resources, and potentially bringing drugs to market faster and with fewer side effects.

### 3.2. ADMET Profiles of the Main Compounds of Tarragon

The ADMET profiles obtained for the compounds identified in higher amounts in tarragon essential oils revealed that all compounds have a good human intestinal absorption. This is not an unexpected result, as monoterpenes usually have small molecular weights and a lipophilic character, and these properties facilitate the intestinal absorption. This result is also in good correlation with other published studies, which reveal the good intestinal absorption and bioavailability of numerous monoterpenes [23,46,47].

In the case of distribution profiles, there are quite different predictions obtained using the two calculation tools, which may be due to the approaches and/or models that are used by the two tools when calculating the predictions. The output of admetSAR3.0 indicates that investigated compounds are not inhibitors of P-gp, whereas the results of ADMETLab3.0 tools emphasize that, except for limonene and eugenol, the other compounds reveal at least low probabilities of inhibiting P-gp. The literature data emphasize that terpenoids are considered to possess significant P-gp inhibitory activity [48]. There also are few inconsistences in the predictions obtained using the two computational tools regarding the inhibition of other membrane transporters (BCRP, BSEP, and MRP1) by the investigated compounds. No studies were found in the specialized literature on the inhibition of BCRP, MATE1, and BSEP transporters by the compounds identified in tarragon essential oils, but the inhibition of these transporters deserves to be exploited through experimental studies, not only because of the inconsistencies in the obtained predictions, but especially because of the importance of inhibiting these transporters. The inhibition of BCRP, MRP1, and P-gp proteins that pump chemotherapeutic drugs out of cells, reducing their effectiveness, leads to preventing the expelling of drugs from the cells, which can increase the intracellular concentration of chemotherapeutic agents, making these compounds more effective against resistant cancer cells.

Another difference appears in the predictions regarding the ability of compounds found in tarragon essential oils to cross the blood–brain barrier. admetSAR3.0 predicts that all these compounds are able to cross the BBB, while ADMETLab3.0 indicates that only limonene, eugenol acetate, and caryophyllene oxide reveal at least low probabilities to cross the blood–brain barrier. A previous computational study performed by our team and based on ADMETLab2.0 indicated that nine acyclic monoterpenes (i.e., beta-myrcene, beta-ocimene, citronellal, citronellol, citronellyl acetate, geranial, geraniol, linalool, and linalyl acetate) revealed high probabilities to penetrate the blood–brain barrier [23]. Predictions of the ability of monoterpenes to cross the BBB are in agreement with published data, revealing that numerous terpenoids demonstrated the ability to cross BBB, and those having small molecular size and high lipophilicity exhibited the highest BBB penetration [49,50,51].

Predictions regarding the metabolism of the investigated compounds reveal that these compounds may be substrates and/or inhibitors of numerous cytochromes. Some of these predictions are in good correlation with published data. For example, a study made by Alharbi and his coworkers showed that eugenol has the ability to inhibit CYP1A2, CYP2C9, CYP2D6, and CYP3A4 [52]. A previous study implemented by our team revealed that several acyclic monoterpenes may inhibit CYP2B6, beta-ocimene and beta-myrcene may be substrates of CYP2C19, geranial may be a substrate for both CYP2C9 and CYP2C19, and beta-ocimene and citronellyl acetate may be inhibitors of CYP1A2 [23]. Taking into account that cytochrome P450 is a family of enzymes involved in the metabolism of various endogenous and exogenous compounds, its inhibition by the compounds found in tarragon essential oil can have significant implications for drug metabolism, potentially causing increased drug levels and toxicity, and there also are potential herb–drug interactions.

There are few predicted toxic effects for the several compounds found in tarragon essential oils: skin sensitization, nephrotoxicity, respiratory toxicity, and carcinogenicity. The prediction regarding the skin sensitization potential of these compounds is in good correlation with published data revealing that plants from the Asteraceae family are capable of leading to this effect [20], and with the results of our previous computational study revealing the skin-sensitizing potential of several acyclic monoterpenes, including beta-ocimene [23]. The respiratory toxicity of acyclic monoterpenes was also predicted in our previous study [23], but experimental studies confirming the respiratory toxicity of the investigated compounds were not identified. As far as we know, no studies on the nephrotoxicity of these compounds can be found in the specialized literature. The carcinogenic effect of estragole and of its metabolites has been noticed in rodents [17,18,53] and the European Food Safety Authority classified estragole and methyl eugenol as genotoxic and carcinogenic compounds [15].

The Food and Drug Administration considers *A. dracunculus* and the extracts and essential oils obtained from this species as safe for use in dietary intake and medical applications [54], but in high concentrations and/or under particular circumstances, they can become toxic [46]. Due to their fragrant character, these compounds are often used in cosmetics and household products, and as a result, human exposure to these compounds is increased. Consequently, the toxicity potential of the compounds found in tarragon extracts (and particularly in those that are concentrated, such as essential oils) should be taken into consideration, especially for individuals who might be professionally exposed to these extracts, such as herbalists, manufacturers of herbal products, or even individuals working in research or pharmaceutical development environments.

Despite the significant advances in computational models and in silico predictions for ADMET properties, these predictions may not always fully reflect real-world results. One of the limitations refers to the accuracy of prediction that depends on the similarity of the properties of the investigated compounds with those of the molecules used for prediction models and/or to the fact that many of the ADMET prediction models are based to in vitro data. Other limitations are due to the fact that these predictions usually do not take into account the concentration of the analyzed compound, its interactions with other compounds, and oversimplify the physiological process, while the biological systems are highly complex. This underlines that experimental validation is required to confirm the computational predictions.

### 3.3. Antibacterial Activity of the Main Compounds of Tarragon Essential Oil

The present study reveals that compounds identified in tarragon essential oils may be effective against several types of microorganisms, including *Staphylococcus*, *Streptococcus*, and *Prevotella* species. Considering that all the mentioned microorganisms can cause several types of human infections, this information becomes valuable. Even though the antibacterial properties of tarragon essential oil are mentioned in many published studies, there were no identified publications that mention that the compounds present in these extracts can be effective against the microorganisms predicted using AntiBac-Pred. For example, one of the published papers reveals that in vitro *tests* showed that *Artemisia* oils are effective against *Candida albicans* [55]. Antibiotics used to treat infections caused by *Staphylococcus*, *Streptococcus*, and *Prevotella* microorganisms are generally effective, but they can also come with a range of side effects that depend on the specific antibiotic class used and the patient’s condition. It is widely recognized that antibiotics cause adverse effects, with common side effects including allergic reactions and gastrointestinal effects, in addition to increased bacterial resistance [56]. Terpenoids and essential oils are among the most common classes of phytochemicals with antibacterial activity [57]. There may be synergistic effects between terpenoids and antibiotics, as terpenoids can enhance the efficacy of antibiotics by increasing the permeability of the bacterial cell membrane [58]. Consequently, the combination of tarragon essential oils and antibiotics may lead to more potent antibacterial effects, reducing the required dose of antibiotics against *Staphylococcus*, *Streptococcus*, and *Prevotella* species, eliminating bacterial resistance, and reducing antibiotic side effects. Further research would be beneficial to confirm the efficacy of the investigated compounds against these specific strains. Considering the current interest of scientists to find natural compounds with antibacterial properties, the potential antimicrobial effect of tarragon extracts should be considered.

### 3.4. Cytotoxicity of the Main Compounds of Tarragon Essential Oils

Compounds identified in higher amounts in the investigated tarragon essential oils are predicted to produce toxicity on numerous types of cancer cells. Several of these predictions are in good agreement with information found in published scientific articles. It was shown that tarragon essential oil had cytotoxic effects on skin melanoma, prostate carcinoma, colorectal adenocarcinoma, gastric adenocarcinoma, and on breast cancer [21,59,60]. Estragole was effective in vitro for inducing apoptosis in breast cancer cells [61]. α-pinene exhibited anticancer activity in ovarian cancer cells by controlling cell multiplication, cell sequence progression, and stimulating apoptosis [62]. D-limonene revealed anticancer properties in in vivo studies concerning breast and pancreatic cancers [31], lung cancer [63], and skin melanoma [32]. Furthermore, limonene was found to enhance cisplatin’s cancer-inhibiting effects in both breast and bladder cancer cell lines [64]. Even though the predicted cytotoxicity of the investigated compounds against different cancer cell lines is promising, it should be noted that these findings are based on computational models and experimental validation is required to confirm this biological activity.

### 3.5. Molecular Docking Analysis

The results obtained using molecular docking reveal that the compounds found in tarragon essential oils may act as moderate inhibitors of RGS17 and have potential clinical utility as chemotherapeutics in the treatment of several types of cancers. It is known that RGS17 regulates various signaling pathways that control cell survival, growth, and invasion. Also, RGS17 plays a role in several cellular processes, and it has been linked to certain diseases, including cancer, neurological disorders, and inflammation [65]. By inhibiting RGS17, the balance of these signaling pathways could be shifted, potentially limiting the ability of cancer cells to proliferate and invade surrounding tissues by reducing cell division, potentially slowing down tumor growth, decreasing cell survival, and increasing apoptosis in tumor cells [66]. Further research is required to assess if the compounds identified in tarragon essential oil are selective inhibitors of RGS17.

## 4. Materials and Methods

### 4.1. Tarragon Extracts

In this study, the following extracts of tarragon were considered for identifying the main chemical compounds: (i) a tarragon essential oil found on the Romanian market (eot-c); (ii) a microwave hexane extraction of shredded dried aerial parts of tarragon (eot-mw); (iii) a hydrodistillate obtained from dried tarragon (eot-hd1); and (iv) a hydrodistillate obtained from fresh tarragon (eot-hd2).

For the extraction of volatile compounds in hexane with the Microwave-Advance System Ethos X (Milestone Srl, Sorisole, Bergamo, Italy) (eot-mw), 3 g of tarragon in 20 mL of hexane was used, and the extraction time was 50 min at 35W on each vessel. Microwave extractions used a 10 min ramp to a 100 °C temperature, 90 bar, and a 30 min hold at this temperature. During the heating process, the rotor was rotated 360° to ensure even heating of all the samples. When the extraction run was complete, the samples were allowed to cool before opening for analysis. The hexane extract was transferred in vials after filtration through a 0.45 µm membrane for GC injection.

In order to obtain the eot-hd1 extract, 100 g of dried aerial parts of the tarragon, which was originally from Iran and found on the Romanian market, was subjected to hydrodistillation using the Clevenger extractor. The resulting essential oils and aromatic water mixture were separated using a separating funnel. The pure essential oils were stored in glass vials at +4 °C until further analysis. The plant oil yields (*w*/*w*) were 0.1%.

For obtaining the eot-hd2 extract, 100 g of fresh cut tarragon was bought on the local market (Timisoara, Romania), and the plant matter was grinded very softly and further subjected to hydrodistillation (105 °C) using the Clevenger extractor. Two solutions were obtained, the distillate, consisting of water and essential oils, and the solution, formed by the condensation of steam in the flask with the plant matter. The two solutions were separated using a separating funnel. The eot-hd2 extract was left to cool and stored at +4 °C until further analysis. The yield of the essential oil was 0.1%.

### 4.2. Identification of Volatile Compounds in Tarragon Essential Oils

The tarragon essential oils were analyzed by a gas chromatography–mass spectrometry method (GC-MS) using a Shimadzu QP 2010 Plus instrument (Columbia, SC, USA). This was achieved by dissolving 20 µL of the tarragon essential oil in 1480 µL of hexane. From the resulting solution, a volume of 1 µL was injected into the GC apparatus at a temperature of 250 °C. The separation of the compounds in the hexane solutions was conducted on an AT-5MS capillary column, with a length of 30 m, a diameter of 0.32 mm, and a layer thickness of 0.25 µm. The separation conditions were as follows: an initial temperature of 40 °C was maintained for 2 min, after which a temperature increase to 250 °C was initiated at a rate of 4 °C/min, followed by a further increase to 300 °C at a rate of 10 °C per minute, where it was maintained for five minutes. The mobile phase was helium 6.0, with a flow rate of 1.92 mL/min. The interface temperature was set to 250 °C, while the ion source temperature was maintained at 210 °C. The identification of the separated compounds was conducted through mass spectrometry, using MS scan mode acquisition (35–500 *m*/*z*) and the NIST database, while their quantification was performed through the normalized area method on SIM mode acquisition. A concordance of at least 90% was observed between the detected compounds and the database. The results were expressed as a percentage of the total number of compounds. The linear retention index (LRI) was calculated using the normal alkane RI for the same type of column.

### 4.3. Prediction of ADME Profiles and Human Health Effects of the Compounds Identified in Higher Amounts in the Obtained Tarragon Essential Oils

To predict the ADME profiles and human health effects of chemical compounds identified in higher concentrations in the obtained tarragon essential oils, the following computational free accessible web services were considered: (i) admetSAR3.0 [28] and ADMETLab3.0 [29] for predicting and comparing the outcomes regarding the ADME profiles and toxicological endpoints; (ii) AntiBac-Pred for predicting the antibacterial effects [30]; and (iii) CLC-Pred2.0 for predicting cell line toxicity [34].

AdmetSAR3.0 is the latest version of the admetSAR tool that provides enhanced prediction accuracy compared to earlier versions. It is used for predicting the ADMET profiles of chemical compounds. The admetSAR3.0 web server hosts over 370,000 manually curated high-quality experimental ADMET annotation data points for 104,652 unique chemical compounds, sourced from the scientific literature and public databases, and incorporates numerous prediction models based on a variety of chemical descriptors and machine learning techniques. The accuracy of admetSAR3.0 predictions varies between 75% and 85% and depends on the specific property being predicted, as different models within the system may have different performance levels for different types of predictions. The chemical structures may be uploaded in several formats and the server provides predictions for 119 endpoints [28].

ADMETLab3.0 is also the latest version of the ADMETLab computational tool that uses quantitative structure–activity relationship (QSAR) models and machine learning algorithms to make ADMET predictions. There are 77 predictive models (18 regression models with R2 values ranging from 0.75 to 0.95, and 59 classification models with the accuracy ranging from 0.72 to 0.99) that take into account diverse chemical features, such as molecular descriptors and structural information, and are trained on large, publicly available datasets of chemical compounds and their ADMET properties. ADMETLab3.0 provides predictions for 21 physicochemical properties, 19 medicinal chemistry properties, 34 ADME endpoints, 36 toxicity endpoints, and 8 toxicophore rules [29].

AntiBac-Pred is a computational tool used to predict the antibacterial activity of chemical compounds, allowing for the classification of chemical structures under investigation into growth inhibitors or noninhibitors of 353 different bacteria strains (both resistant and nonresistant ones) with an accuracy ranging from 0.92 to 0.94. It uses machine learning and cheminformatics approaches to predict if a chemical compound might inhibit the growth of bacteria or act as an antimicrobial agent using antibacterial activity data available in the ChEMBL database. Prediction is obtained by evaluating the chemical structure against the training set and two probabilities are calculated for the ability of the compound to inhibit the growth of the particular bacteria strain, i.e., the probability for the compound to be active (Pa) and the probability for the compound to be inactive (Pi), respectively. The prediction provided by AntiBac-Pred is the confidence, computed as the difference between Pa and Pi [30]. In the present study, a confidence higher than 0.5 is used as a threshold to categorize compounds as potential inhibitors of the growth of bacteria.

The Cell Line Cytotoxicity Predictor version 2 (CLC-Pred2.0) is a computational tool that predicts the cytotoxicity of various compounds across 47 normal human cell lines and 391 cancer cell lines with a mean accuracy ranging from 0.923 to 0.925. It also has the ability to predict the molecular mechanisms of action leading to cytotoxicity by predicting the molecular targets of the analyzed compounds, which significantly increases the possibility of finding anticancer compounds. The machine learning models used by CLC-Pred2.0 are built using a large dataset of known cytotoxicity information extracted from PubChem and ChEMBL databases and statistical learning methods are considered to establish relationships between compound characteristics and cytotoxicity. The machine learning algorithms predicts the probability that a compound is active (cytotoxic in this case) based on its molecular structure and other descriptors. The probability to be active (Pa) is expressed as a score between 0 and 1, with a score closer to 1 suggesting a higher likelihood of the compound being cytotoxic [34]. In the present study, Pa > 0.5 is used as a threshold to categorize compounds as cytotoxic.

### 4.4. Molecular Docking Study

Molecular docking is a molecular modeling method largely used to predict how a bioactive compound might interact with specific biological targets, as it provides insights into the binding affinity of compounds to their targets. In this study, molecular docking has been considered for assessing the possible interactions of the compounds found in tarragon extracts with the RGS17 protein. The SwissDock server has been considered for implementing docking studies [67,68] using AutoDockVina1.2.0 [69] as the docking method, with a box size of 30 × 30 × 30 Å^3^ (a dimension that covers the entire potential binding region) and sampling exhaustivity of 30 (meaning a medium exhaustive search, in which the algorithm finds the best binding pose within a reasonable amount of time). The docking results were visualized using Chimera1.16 software [27]. SwissDock has been considered for implementing molecular docking as it is completely free to use, provides an intuitive interface, and robust docking with energy-based methods. AutoDock Vina, as a docking method, offers faster performance and excellent scoring functions.

## 5. Conclusions

Tarragon’s medicinal use is mainly based on traditional medicine and preliminary scientific studies. The present computational study reveals that compounds found in essential oils of tarragon that is commercialized on the Romanian market have acceptable ADMET profiles, but several compounds may induce skin sensitization and respiratory toxicity, and all of them are potential inhibitors of organic anion transporters OATP1 and OATP2. These toxicity risks should be taken into consideration when people are exposed to high concentrations or use the tarragon essential oils over prolonged periods. Several of the investigated compounds were predicted to exert antibacterial properties against some of the *Staphylococcus*, *Streptococcus*, and *Prevotella* species and may be toxic against several cancer cell types. All these findings may guide further experimental studies for identifying medical applications of the tarragon essential oil or of specific compounds that may be isolated from these extracts.

## Figures and Tables

**Table 1:** Profile of compounds identified in tarragon essential oils: eot-c—commercial tarragon essential oil, eot-mw—essential oil obtained after microwave hexane extraction of shredded dried tarragon, eot-hd1—essential oil obtained after the hydrodistillation of shredded dried tarragon, eot-hd2—essential oil obtained after the hydrodistillation of shredded fresh leaves of tarragon, MH—hydrogenated monoterpenes, MO—oxygenated monoterpenes, SH—hydrogenated sesquiterpenes, SO—oxygenated sesquiterpenes, O—other, and LRIc/LRIr—linear retention index calculated/reference.

Compound	Type	LRIc/ LRIr [25]	eot-c %	eot-mw %	eot-hd1 %	eot-hd2 %
α-Pinene	MH	923/925	1.002	0.417	0.371	-
Camphene	MH	938/940	0.079	-	-	-
Sabinene	MH	960/963	0.088	0.142	0.132	0.232
β-Pinene	MH	975/980	0.133	0.182	0.170	
β-Myrcene	MH	990/992	0.161			
*p*-Cymol	MH	1010/1015	-	0.156	0.135	
Limonene	MH	1020/1023	3.135	2.376	2.137	0.183
cis-β-Ocimene	MH	1035/1038	8.422	1.740	1.567	0.325
trans-β-Ocimene	MH	1042/1045	6.690	2.299	2.089	0.481
β-Linalool	MO	1090/1095	-	-	-	0.641
allo Ocimene	MH	1125/1126	0.219	-	-	-
Estragole	MO	1180/1178	79.425	80.096	82.095	63.148
Carvone	MO	1235/1240	-	0.213	0.214	0.693
α-Citral	MO	1245/1250	-	-	-	-
Bornyl acetate	MO	1265/1268	0.092	0.314	0.312	0.205
Anethole	MO	1280/1285	-	0.177	0.151	-
Eugenol	MO	1350/1352	-	1.977	1.327	-
Methyl cinnamate	MO	1360/1362	-	0.174	0.150	-
Methyl eugenol ether	MO	1401/1402	0.378	4.028	4.017	33.971
Caryophyllene	SH	1410/1408	0.068	0.593	0.640	-
γ-decalactone	O	1448/1450	-	0.261	0.204	-
α-trans-Bergamotene		1460/1458	0.070	0.117	0.113	-
Eugenol acetate	MO	1520/1523	-	2.048	1.896	-
β-Sesquiphellandrene	SH	1528/1530	-	0.216	0.176	-
Caryophyllene oxide	SO	1575/1580	-	2.473	2.106	-
MH			19.829	7.312	6.601	1.221
MO			79.825	89.027	90.162	98.658
SH			0.068	0.809	0.814	
SO				2.473	2.106	
O				0.261	0.204	

**Table 2:** Toxicity profiles obtained using the admetSAR3.0 tool for the compounds identified in higher amounts in obtained tarragon essential oils: NT—neurotoxicity, hERG—cardiotoxicity, RT—respiratory toxicity, NphT—nephrotoxicity, SSens—skin sensitization, Ames—Ames mutagenesis, RepT—reproductive toxicity, and HemT—hemolytic toxicity. Red cells emphasize higher probabilities, orange cells emphasize reasonable probabilities, and yellow cells emphasize low probabilities of these compounds to produce the predicted toxicity.

Compound/Toxicity	NT	hERG 10–30 µM	RT	NphT	SSens	Ames	RepT	HemT
Estragole	−2.295	0.256	0.143	0.893	0.839	0.240	0.223	0.166
α-Pinene	−2.633	0.465	0.477	0.704	0.923	0.074	0.172	0.361
cis-β-Ocimene	−2.537	0.569	0.269	0.629	0.971	0.098	0.063	0.407
trans-β-Ocimene	−2.536	0.556	0.272	0.626	0.971	0.098	0.062	0.408
Limonene	−2.618	0.565	0.275	0.592	0.916	0.069	0.095	0.424
Eugenol methyl ether	−2.203	0.379	0.121	0.846	0.684	0.308	0.195	0.101
Eugenol acetate	−2.542	0.470	0.079	0.711	0.476	0.452	0.235	0.054
Eugenol	−2.175	0.132	0.189	0.496	0.809	0.123	0.213	0.125
Caryophyllene oxide	−2.608	0.836	0.500	0.690	0.913	0.137	0.167	0.342

**Table 3:** Toxicity profiles obtained using the ADMETLab3.0 tool for the compounds identified in higher amounts in obtained tarragon essential oils: hERG—cardiotoxicity, H-HT—hepatotoxicity, Ames—Ames mutagenicity, SSens—skin sensitization, RT—respiratory toxicity, NT—neurotoxicity, NphT—nephrotoxicity, and HemT—hemolytic toxicity. Red cells emphasize higher probabilities, orange cells emphasize reasonable probabilities, and yellow cells empha-size low probabilities of these compounds to produce the predicted toxicity.

Compound /Toxicity	hERG 10 µM	H-HT	Ames	SSens	Carcinogenicity	RT	NT	NphT	HemT
Estragole	0.695	0.315	0.502	0.881	0.644	0.814	0.180	0.673	0.174
α-Pinene	0.796	0.331	0.016	0.938	0.629	0.844	0.008	0.655	0.124
cis-β-Ocimene	0.632	0.437	0.337	0.963	0.567	0.796	0.142	0.674	0.275
trans-β-Ocimene	0.470	0.624	0.510	0.928	0.499	0.827	0.307	0.544	0.372
Limonene	0.344	0.401	0.192	0.920	0.691	0.637	0.436	0.136	0.384
Eugenol methyl ether	0.625	0.345	0.468	0.880	0.649	0.815	0.172	0.702	0.214
Eugenol acetate	0.479	0.314	0.461	0.940	0.470	0.690	0.083	0.600	0.272
Eugenol	0.658	0.310	0.468	0.930	0.582	0.822	0.138	0.563	0.136
Caryophyllene oxide	0.540	0.476	0.556	0.815	0.715	0.621	0.208	0.618	0.416

**Table 4:** Predictions regarding the antibacterial properties of the main compounds of tarragon essential oils. The confidence of each prediction is given in the parentheses.

Compound	Antibacterial Property
cis-β-Ocimene and trans-β-Ocimene	*Staphylococcus simulans* (0.713), *Streptococcus mutans* (0.509)
α-Pinene	*Resistant Staphylococcus simulans* (0.714), *Staphylococcus sciuri* (0.593), *Staphylococcus simulans* (0.539)
Caryophyllene oxide	*Prevotella melaninogenica* (0.556), *Prevotella intermedia* (0.509)

**Table 5:** Cytotoxicity, predicted molecular targets, and the effects on these targets of the main compounds found in obtained tarragon essential oils. The probability that the compound is active against the cell is given in parentheses.

Compound	Cytotoxicity	Molecular Target and the Type of Effect the Chemical May Have on the Target
Estragole	cisplatin-resistant ovarian carcinoma (0.786), diffuse large B-cell lymphoma activated B-cell type (0.616), skin melanoma (0.568), bronchioalveolar carcinoma (0.551), papillary adenocarcinoma (0.500)	regulator of G-protein signaling 17 inhibitor (0.523)
cis-β-Ocimene and trans-β-Ocimene	cisplatin-resistant ovarian carcinoma (0.883), diffuse large B-cell lymphoma activated B-cell type (0.610), amelanotic melanoma (0.563), skin melanoma (0.534)	N-arachidonoyl glycine receptor agonist (0.943), regulator of G-protein signaling 17 inhibitor (0.848), DNA polymerase kappa inhibitor (0.747), serine/threonine-protein phosphatase 2A 56 kDa regulatory subunit alpha isoform inhibitor (0.737), emopamil-binding protein-like inhibitor (0.703), bile salt export pump inhibitor (0.662), nuclear receptor subfamily 1 group I member 2 antagonist (0.655), sarcoplasmic/endoplasmic reticulum calcium ATPase 3 inhibitor (0.637), ATP-dependent DNA helicase Q1 inhibitor (0.625), RecQ-like DNA helicase BLM inhibitor (0.624)
Limonene	cisplatin-resistant ovarian carcinoma (0.808), skin melanoma (0.566)	N-arachidonoyl glycine receptor agonist (0.874), regulator of G-protein signaling 17 inhibitor (0.780), DNA polymerase kappa inhibitor (0.649), thyrotropin receptor agonist (0.627), bile salt export pump inhibitor (0.577), cytochrome 2A13 inhibitor (0.512)
α-Pinene	cisplatin-resistant ovarian carcinoma (0.841)	C-X-C chemokine receptor type 3 antagonist (0.970), regulator of G-protein signaling 17 inhibitor (0.511)
Eugenol methyl ether	cisplatin-resistant ovarian carcinoma (0.760), skin melanoma (0.616), diffuse large B-cell lymphoma activated B-cell type (0.579), bronchioalveolar carcinoma (0.544)	polyunsaturated fatty acid lipoxygenase ALOX12 inhibitor (0.517), regulator of G-protein signaling 17 inhibitor (0.502)
Eugenol acetate	cisplatin-resistant ovarian carcinoma (0.825), bronchioalveolar carcinoma (0.548)	DNA polymerase kappa inhibitor (0.573), mitogen-activated protein kinase 1 inhibitor (0.549), polyunsaturated fatty acid lipoxygenase ALOX12 inhibitor (0.548), cytochrome 2C19 inhibitor (0.518), RecQ-like DNA helicase BLM inhibitor (0.505), polyunsaturated fatty acid lipoxygenase ALOX15 inhibitor (0.500)
Eugenol	cisplatin-resistant ovarian carcinoma (0.788), skin melanoma (0.576), bronchioalveolar carcinoma (0.514), clear cell renal cell carcinoma (0.503)	polyunsaturated fatty acid lipoxygenase ALOX12 inhibitor (0.719), polyunsaturated fatty acid lipoxygenase ALOX15 inhibitor (0.690), regulator of G-protein signaling 17 inhibitor (0.589), RecQ-like DNA helicase BLM inhibitor (0.536), cytochrome 2C19 inhibitor (0.514)
Caryophyllene oxide	promyeloblast leukemia (0.819), cisplatin-resistant ovarian carcinoma (0.762), breast adenocarcinoma (0.720), brain glioma (0.707), breast carcinoma (0.692), skin melanoma (0.685), renal carcinoma (0.669), non-small cell lung carcinoma (0.631), gastric carcinoma (0.617), ovarian adenocarcinoma (0.612), pancreatic carcinoma (0.606), non-small cell lung carcinoma (0.605), lung carcinoma (0.602), breast carcinoma (0.592), renal cell carcinoma (0.587), colon adenocarcinoma (0.579), amelanotic melanoma (0.578), breast ductal carcinoma (0.575), adult immunoblastic lymphoma (0.553), astrocytoma (0.522), multiple myeloma (0.513)	regulator of G-protein signaling 17 inhibitor (0.276)

**Table 6:** Interacting energies and amino acids involved in the interactions between the compound found in the obtained tarragon essential oils and the regulator of G-protein signaling 17.

Compound	Without Ca^2+^		In Presence of Ca^2+^	
	ΔG (kcal/mol)	Amino Acids Involved in the Interaction	ΔG (kcal/mol)	Amino Acids Involved in the Interaction
Estragole	−4.916	hydrophobic interactions with TRP114, GLN175, LEU176, π stacking with TYR179	−5.029	hydrophobic interactions with TRP114, GLN175, LEU176, TYR179, π stacking with TYR179
α-Pinene	−4.810	hydrophobic interactions with TRP114, GLN175, LEU176, TYR179	−4.821	hydrophobic interactions with ALA116, ASP119, LYS132, MET135, ALA136
cis-β-Ocimene	−4.866	hydrophobic interactions with MET89, LYS90, TRP114, GLN175, LEU176, TYR179	−4.944	hydrophobic interactions with TRP114, GLN175, LEU176, TYR179
trans-β-Ocimene	−4.959	hydrophobic interactions with ASP86, MET89, LYS90, TRP114, GLN175, LEU176, TYR179	−5.339	hydrophobic interactions with MET89, LYS90, TRP114, GLN175, LEU176, TYR179
Limonene	−5.278	hydrophobic interactions with ASP86, MET89, TRP114, GLN175, LEU176, TYR179	−4.937	hydrophobic interactions with TRP114, GLN175, LEU176, TYR179
Eugenol methyl ether	−5.074	hydrophobic interactions with TRP114, GLN175, LEU176, π stacking with TYR179	−4.995	hydrophobic interactions with TRP114, GLN175, LEU176, TYR179, π stacking with TYR179
Eugenol acetate	−5.539	hydrophobic interactions with ASP86, MET89, LYS90, TRP114, GLN175, LEU176, π stacking with TYR179	−5.423	hydrophobic interactions with MET89, LYS90, TRP114, GLN175, LEU176, TYR179, π stacking with TYR179
Eugenol	−5.232	hydrophobic interactions with GLN175, LEU176, TYR179, π stacking with TYR179	−4.949	hydrophobic interactions with TRP114, GLN175, LEU176, TYR179, π stacking with TYR179
Caryophyllene oxide	−3.681	hydrophobic interactions with LEU115, ALA116, ASP119, MET135, ILE136, TYR140	−3.971	hydrophobic interactions with ALA116, LYS132, MET135, ILE136, TYR140

## Data Availability

All data are presented in the manuscript and Supplementary Materials.

## Supplementary Materials

The following supporting information can be downloaded at: https://www.mdpi.com/article/10.3390/ijms26051860/s1.

## Abbreviations

The following abbreviations are used in this manuscript:

ADME	Absorption, distribution, metabolism, excretion
ADMET	Absorption, distribution, metabolism, excretion, toxicity
Ames	Ames mutagenesis
BBB	blood–brain barrier
BCRP	breast cancer resistance protein
BSEP	bile salt export pump
CLp	plasmatic clearance
CYP	cytochrome P
LRIc/LRIr	linear retention index calculated/reference
HemT	hemolytic toxicity
hERG	Cardiotoxicity
MATE1	multidrug and toxin extrusion protein 1
MH	hydrogenated monoterpenes
MO	oxygenated monoterpenes
MRP1	multidrug resistance protein 1
NphT	Nephrotoxicity
NT	neurotoxicity
P-gp	P glycoprotein
QSAR	quantitative structure–activity relationship
RepT	reproductive toxicity
RT	respiratory toxicity
SH	hydrogenated sesquiterpenes
SO	oxygenated sesquiterpenes
SSens	skin sensitization
