# Supplementary material for: Predictions of the Biological Effects of the Main Components of Tarragon Essential Oil
Source: Int J Mol Sci. 2025 Feb 21;26(5):1860. doi: 10.3390/ijms26051860 (PMC11899843; doi:10.3390/ijms26051860)
Supplement: Supplementary file 1 [file ijms-26-01860-s001.zip › ijms-3485304-supplementary.pdf]

Predictions of the biological effects of the main components of tarragon essential oil

Andrijana Pujicic[1], Iuliana Popescu[2], Daniela Dascalu [3], David Emanuel Petreuș[3], Adriana Isvoran[1]

[1]Department of Biology, West University of Timisoara, 16 Pestalozzi, 300115 Timișoara, Romania, andrijana.pujicic@e-uvt.ro

[2]Faculty of Agriculture, University of Life Sciences ”King Mihai I” from Timisoara, 119 Calea Aradului, 300645 Timişoara, Romania, iuliana_popescu@usvt.ro

[3]Department of Chemistry, West University of Timisoara, 16 Pestalozzi, 300115 Timișoara, Romania; david.petreus03@e-uvt.ro

* Correspondence: adriana.isvoran@e-uvt.ro

Supplementary material

Chromatogram eo001tarhon D:\ANALIZE GC\2023\teste MC\eo001tarhon_UNK-0001_13.03.2024_1.qgd

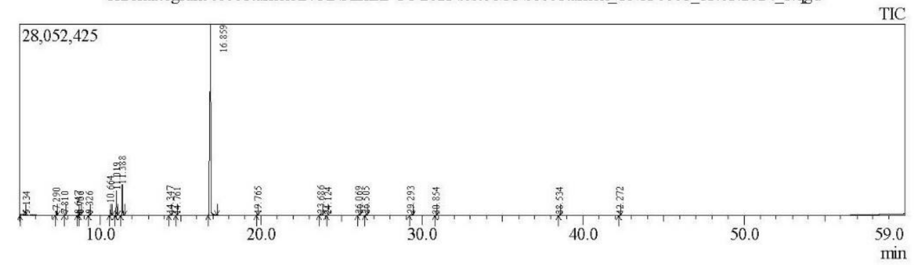

Quantitative Result Table

ID#	Name	R.Time	m/z	Area	Height	Conc.
1	.alpha.-Pinene	7.290	TIC	1485067	590084	1.002
2	Camphene	7.810	TIC	117232	40677	0.079
3	Sabinene	8.647	TIC	130400	47311	0.088
4	.beta.-Pinene	8.766	TIC	197148	73318	0.133
5	.beta.-Myrcene	9.326	TIC	238579	90370	0.161
6	D-Limonene	10.664	TIC	4645492	1667297	3.135
7	trans-.beta.-Ocimene	11.019	TIC	9911369	3557292	6.690
8	cis-.beta.-Ocimene	11.388	TIC	12477514	4456610	8.422
9	2,4,6-Octatriene, 2,6-dimethyl-, (E,Z)-	14.347	TIC	324078	84796	0.219
10	2-Methyl-1-nonene-3-yne	14.761	TIC	57789	17850	0.039
11	Estragole	16.859	TIC	117675185	27932629	79.425
12	Bornyl acetate	19.765	TIC	137010	50252	0.092
13	Eugenol methyl ether	23.686	TIC	559616	131618	0.378
14	Caryophyllene	24.124	TIC	99936	27562	0.067
15	trans-.alpha.-Bergamotene	26.505	TIC	102314	32131	0.069

Figure S1. Chromatogram obtained for the commercial essential oil of tarragon (eot-c)

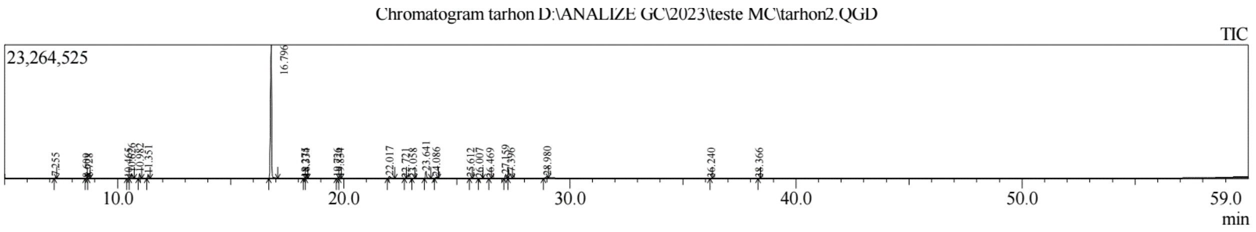

Quantitative Result Table

ID#	Name	R.Time	m/z	Area	Height	Conc.	Conc.Un
1	.alpha.-Pinene	7.255	TIC	432672	164996	0.417 %	
2	Sabinene	8.609	TIC	147442	55671	0.142 %	
3	.beta.-Pinene	8.728	TIC	188721	66044	0.182 %	
4	p-Cymol	10.465	TIC	161346	55920	0.156 %	
5	D-Limonene	10.626	TIC	2462827	859989	2.376 %	
6	beta.-trans-Ocimene	10.982	TIC	2383186	859016	2.299 %	
7	beta.-cis-Ocimene	11.351	TIC	1804054	634315	1.740 %	
8	Estragole	16.796	TIC	83024579	23177471	80.096 %	
9	-Carvone	18.334	TIC	221239	66941	0.213 %	
10	Bornyl acetate	19.726	TIC	325303	107889	0.314 %	
11	Anethole	19.834	TIC	183560	54093	0.177 %	
12	Eugenol	22.017	TIC	2048854	384171	1.977 %	
13	Methyl cinnamate	23.058	TIC	180791	49590	0.174 %	
14	Methyl eugenol ether	23.641	TIC	4175670	1250024	4.028 %	
15	Caryophyllene	24.086	TIC	614673	197749	0.593 %	
16	gamma.-Decalactone	25.612	TIC	270754	68442	0.261 %	
17	trans-.alpha.-Bergamotene	26.469	TIC	120781	34719	0.117 %	
18	Eugenol acetate	27.159	TIC	2122733	586523	2.048 %	
19	beta.-Sesquiphellandrene	27.396	TIC	223463	38310	0.216 %	
20	Caryophyllene oxide	28.980	TIC	2563828	360507	2.473 %	

Figure S2. Chromatogram for the tarragon essential oil obtained by microwave hexane extraction of shredded dried aerial parts of tarragon (eot-mw)

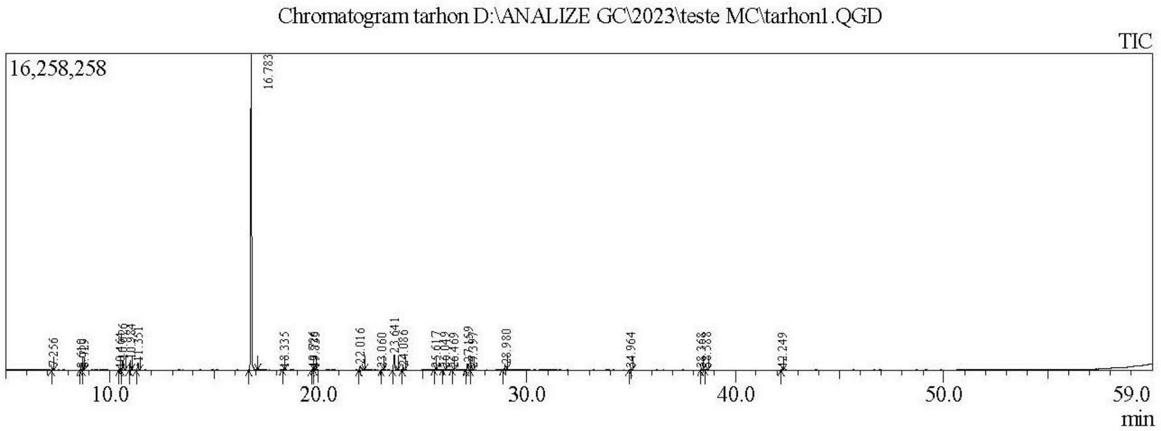

Quantitative Result Table

ID#	Name	R.Time	m/z	Area	Height	Conc.
1	.alpha.-Pinene	7.256	TIC	235877	89514	0.371
2	Sabinene	8.610	TIC	83622	32293	0.132
3	.beta.-Pinene	8.729	TIC	107513	37105	0.169
4	p-Cymol	10.464	TIC	86086	29198	0.135
5	D-Limonene	10.626	TIC	1358260	476398	2.137
6	beta.-trans-Ocimene	10.984	TIC	1328004	475768	2.089
7	beta.-cis-Ocimene	11.351	TIC	995935	350539	1.567
8	Estragole	16.783	TIC	52189946	16199858	82.095
9	-Carvone	18.335	TIC	136144	40178	0.214
10	Bornyl acetate	19.726	TIC	198404	64492	0.312
11	Anethole	19.835	TIC	95754	29681	0.151
12	Eugenol	22.016	TIC	843782	207249	1.327
13	Methyl cinnamate	23.060	TIC	95161	25664	0.150
14	Methyl eugenol ether	23.641	TIC	2553976	780103	4.017
15	Caryophyllene	24.086	TIC	406376	120508	0.639
16	gamma.-Decalactone	25.617	TIC	129728	38089	0.204
17	trans-.alpha.-Bergamotene	26.469	TIC	71743	18440	0.113
18	Eugenol acetate	27.159	TIC	1205172	332578	1.896
19	beta.-Sesquiphellandrene	27.397	TIC	112076	21970	0.176
20	Caryophyllene oxide	28.980	TIC	1338939	197192	2.106

Figure S3. Chromatogram for the tarragon essential oil extracted from the hydrodistilate obtained from dried tarragon (eot-hd1)

Chromatogram vo D:\ANALIZE GC\2023\teste MC\vo_tar-0002_05.06.2024_2.qgd

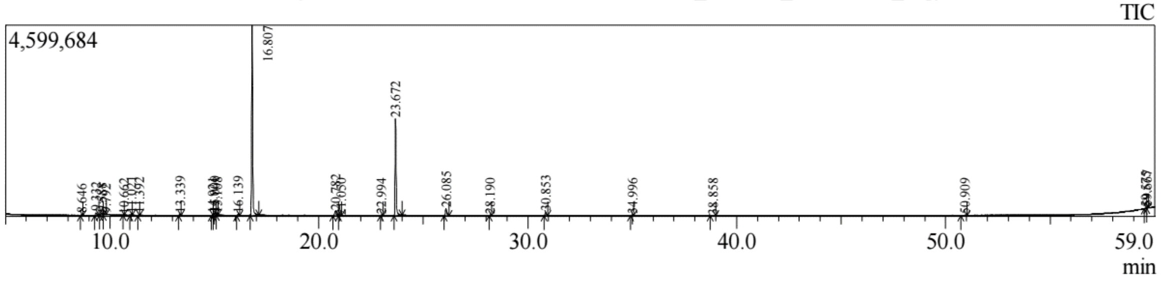

Quantitative Result Table

ID#	Name	R.Time	m/z	Area	Height	Conc.
1	Sabinene	8.646	TIC	55283	18672	0.232
2	Limonene	10.662	TIC	43508	13273	0.183
3	beta.-trans-Ocimene	11.021	TIC	114599	36022	0.481
4	beta.-cis-Ocimene	11.392	TIC	77389	25141	0.325
5	beta.-Linalool	13.339	TIC	152656	37087	0.641
6	4-Carvomenthenol	16.139	TIC	164959	44126	0.693
7	Estragole	16.807	TIC	15029629	4570752	63.148
8	Nerol acetate	22.994	TIC	48825	13839	0.205
9	Eugenol methyl ether	23.672	TIC	8085175	2331759	33.971
10	Elemicin	28.190	TIC	28442	7276	0.120

Figure S4. Chromatogram for the tarragon essential oil extracted from the hydrodistilate obtained from fresh tarragon (eot-hd2)

Table S1. Physico-chemical properties of the compounds identified in higher ammount in obtained tarragon essential oils

Compound / property	MW (g/mol)	nHA	nHD	**nRot**	**logP**
estragole	148.09	1	0	3	2.895
α-pinene	136.13	0	0	0	4.514
cis-β-ocimene	136.13	0	0	3	4.358
trans-β-ocimene	136.13	0	0	3	4.275
limonene	136.13	0	0	1	4.541
eugenol methyl ether	178.10	2	0	4	2.473
eugenol acetate	206.09	3	0	5	2.466
eugenol	164.08	2	1	3	2.321
caryophyllene oxide	220.18	1	0	3	3.024

Table S2. Medicinal chemistry for the compounds identified in higher amount in obtained tarragon essential oils and how well a compound's data fits within the applicability domain (AD) i.e. the training dataset of the predictive models. Green cells illustrate that the rules are met, and red cells illustrate that the rules are not followed.

Compound / property	Lipinski rule	Pfizer rule	GSK rule	AD admetSAR3.0	AD ADMETLab3.0
estragole	Accepted	Accepted	Accepted	50% in AD	In upper limit of AD
α-pinene	Accepted	Rejected	Rejected	50% in AD	In upper limit of AD
cis-β-ocimene	Accepted	Rejected	Rejected	50% in AD	In upper limit of AD
trans-β-ocimene	Accepted	Rejected	Rejected	50% in AD	In upper limit of AD
limonene	Accepted	Rejected	Rejected	50% in AD	In upper limit of AD
eugenol methyl ether	Accepted	Accepted	Accepted	50% in AD	In upper limit of AD
eugenol acetate	Accepted	Accepted	Accepted	50% in AD	In upper limit of AD
eugenol	Accepted	Accepted	Accepted	100% in AD	In upper limit of AD
caryophyllene oxide	Accepted	Rejected	Accepted	50% in AD	In upper limit of AD

Table S3. Absorption and distribution profile of the compounds identified in higher amount in tarragon essential oils and obtained using admetSAR3.0 tool: HIA – human intestinal absorption lower than 30%. BBB- blood brain barrier, OATP1B1 – organic anion transporter peptide 1B1, OATP1B3 – organic anion transporter peptide 1B3, OATP2B1 – organic anion transporter peptide 2B1, OCT1 – organic cation transporter 1, OCT2 – organic cation transporter 2, BCRP – breast cancer resistance protein, BSEP - bile salt export pump, MATE1 - multidrug and toxin extrusion protein 1, Pgp – P-glycoprotein, PPB – plasma protein binding. Red cells emphasize higher probabilities, and yellow cells emphasize low probabilities of these compounds to produce the predicted toxicity.

Compound / property	HIA <30%	BBB	OATP1B1 inhibitor	OATP1B3 inhibitor	OATP2B1 inhibitor	OCT1 inhibitor	OCT2 inhibitor	BCRP onhibitor	BSEP inhib	MATE1 inhib	Pgp inhibitor	Pgp substrate	PPB
estragole	0.985	0.991	0.996	0.997	0.049	0.400	0.219	0.088	0.191	0.029	0.010	0.076	0.800
α-pinene	0.965	0.984	0.970	0.977	0.105	0.530	0.377	0.241	0.494	0.043	0.068	0.147	0.810
cis-β-ocimene	0.944	0.969	0.954	0.969	0.131	0.545	0.268	0.308	0.478	0.061	0.102	0.094	0.748
trans-β-ocimene	0.943	0.968	0.954	0.968	0.133	0.544	0.264	0.308	0.477	0.062	0.102	0.095	0.746
limonene	0.950	0.979	0.967	0.973	0.120	0.560	0.408	0.279	0.542	0.064	0.111	0.173	0.739
eugenol methyl ether	0.992	0.994	0.998	0.999	0.033	0.422	0.237	0.073	0.162	0.032	0.018	0.078	0.688
eugenol acetate	0.993	0.993	0.997	0.998	0.043	0.371	0.193	0.100	0.202	0.048	0.026	0.068	0.662
eugenol	0.986	0.952	0.992	0.996	0.058	0.240	0.116	0.057	0.118	0.055	0.012	0.051	0.600
caryophyllene oxide	0.940	0.984	0.968	0.971	0.152	0.705	0.468	0.287	0.678	0.079	0.155	0.181	0.842

Table S4. Absorption and distribution profile of the compounds identified in higher amount in tarragon essential oils and obtained using ADMETLab3.0 tool: HIA – human intestinal absorption lower than 30%. BBB- blood brain barrier, OATP1B1 – organic anion transporter peptide 1B1, OATP1B3 – organic anion transporter peptide 1B3, BCRP – breast cancer resistance protein, MRP1- Multidrug Resistance Protein 1, BSEP - bile salt export pump, Pgp – P-glycoprotein, PPB – plasma protein binding. Red cells emphasize higher probabilities and yellow cells emphasize low probabilities of these compounds to produce the predicted toxicity.

Compound / property	HIA>30%	BBB	OATP1B1 inhibitor	OATP1B3 inhibitor	BCRP inhibitor	MRP1 inhibitor	BSEP inhibitor	Pgp inhibitor	Pgp substrate	PPB (%)
estragole	0.438	0.204	0.998	0.983	0.879	0.123	0.994	0.900	0.001	87.067
α-pinene	0.006	0.406	0.957	0.969	0.076	0.755	0.810	0.695	0.076	74.420
cis-β-ocimene	0.190	0.132	0.955	0.747	0.794	0.852	0.456	0.875	0.098	89.217
trans-β-ocimene	0.113	0.027	0.988	0.758	0.815	0.863	0.732	0.930	0.128	90.312
limonene	0.001	0.984	0.959	0.882	0.276	0.980	0.595	0.407	0.011	91.868
eugenol methyl ether	0.079	0.278	0.996	0.991	0.907	0.250	0.997	0.919	0.003	85.727
eugenol acetate	0.026	0.598	0.999	0.997	0.601	0.309	0.997	0.756	0.003	79.028
eugenol	0.035	0.106	0.999	0.998	0.914	0.418	0.963	0.468	0.002	80.610
caryophyllene oxide	0.003	0.646	0.999	0.993	0.392	0.730	0.977	0.969	0.008	96.268

Table S5. Metabolism and excretion profiles of the compounds identified in higher amount in tarragon essential oils and obtained using admetSAR3.0 tool: CYP – cytochrome P450; CLp – clearance. Red cells emphasize higher probabilities, orange cells emphasize reasonable probabilities, and yellow cells emphasize low probabilities of these compounds to produce the predicted activity.

Compound / property	Inhibitor						substrate						CLp
	CYP1A2	CYP3A4	CYP2B6	CYP2C9	CYP2C19	CYP2D6	CYP1A2	CYP3A4	CYP2B6	CYP2C9	CYP2C19	CYP2D6	
estragole	0.767	0.011	0.539	0.087	0.640	0.174	0.746	0.449	0.966	0.321	0.549	0.372	0.696
α-pinene	0.336	0.015	0.562	0.076	0.428	0.162	0.087	0.066	0.906	0.040	0.065	0.044	0.602
cis-β-ocimene	0.454	0.006	0.691	0.051	0.408	0.177	0.073	0.060	0.881	0.037	0.042	0.060	0.819
trans-β-ocimene	0.450	0.006	0.697	0.051	0.404	0.179	0.075	0.062	0.881	0.038	0.043	0.063	0.823
limonene	0.376	0.016	0.588	0.069	0.410	0.195	0.077	0.058	0.864	0.038	0.050	0.062	0.678
eugenol methyl ether	0.856	0.019	0.431	0.110	0.669	0.234	0.801	0.531	0.963	0.328	0.662	0.516	0.769
eugenol acetate	0.880	0.007	0.375	0.089	0.447	0.097	0.814	0.453	0.875	0.451	0.669	0.584	0.899
eugenol	0.831	0.010	0.332	0.093	0.325	0.257	0.560	0.292	0.678	0.402	0.427	0.386	0.918
caryophyllene oxide	0.447	0.006	0.800	0.060	0.402	0.263	0.420	0.287	0.952	0.133	0.338	0.319	0.636

Table S6. Metabolism and excretion profiles of the compounds identified in higher amount in tarragon essential oils and obtained using ADMETLab3.0 tool. Red cells emphasize higher probabilities, orange cells emphasize reasonable probabilities, and yellow cells emphasize low probabilities of these compounds to produce the predicted activity.

Compound / property	Inhibitor						substrate						CLp mL/min/kg
	CYP1A2	CYP3A4	CYP2B6	CYP2C9	CYP2C19	CYP2D6	CYP1A2	CYP3A4	CYP2B6	CYP2C9	CYP2C19	CYP2D6	
Estragole	0.368	0.995	0.989	0.900	0.991	0.391	0.994	0.069	0.122	0.995	0.989	1.000	11.721
α-pinene	0.004	0.249	0.346	0.514	0.959	0.419	0.253	0.104	0.916	0.860	0.996	0.993	11.097
cis-β-ocimene	0.029	0.104	0.999	0.021	0.990	0.063	0.043	0.022	0.569	0.221	0.985	0.819	11.032
trans-β-ocimene	0.040	0.047	0.986	0.013	0.935	0.046	0.009	0.033	0.106	0.111	0.981	0.809	10.846
limonene	0.593	0.062	0.635	0.218	0.760	0.019	0.074	0.009	0.919	0.986	0.966	0.955	11.953
eugenol methyl ether	0.689	1.000	0.885	0.915	0.959	0.048	0.941	0.017	0.909	1.000	1.000	1.000	7.76
eugenol acetate	0.962	0.832	0.971	0.913	0.998	0.418	0.083	0.000	0.020	0.999	0.954	0.312	6.55
eugenol	0.997	0.998	0.999	0.601	0.993	0.342	0.334	0.000	0.043	1.000	0.991	0.999	10.004
caryophyllene oxide	0.004	0.046	0.946	0.085	0.953	0.030	0.007	0.100	0.131	0.135	0.987	0.330	11.491
